# Improving access to pre-exposure prophylaxis for adolescent girls and young women: recommendations from healthcare providers in eastern Zimbabwe

**DOI:** 10.1186/s12879-022-07376-5

**Published:** 2022-04-23

**Authors:** Morten Skovdal, Phyllis Magoge-Mandizvidza, Freedom Dzamatira, Rufurwokuda Maswera, Constance Nyamukapa, Ranjeeta Thomas, Owen Mugurungi, Simon Gregson

**Affiliations:** 1 Department of Public Health, University of Copenhagen, Øster Farimagsgade 5, 1014 Copenhagen, Denmark; 2 Manicaland Centre for Public Health Research, Biomedical Research and Training Institute, Harare, Zimbabwe; 3 Department of Infectious Disease Epidemiology, Imperial College London, London, UK; 4 Department of Health Policy, London School of Economics and Political Science, London, UK; 5 AIDS and TB Programme, Ministry of Health and Child Welfare, Harare, Zimbabwe

**Keywords:** HIV, Prevention, PrEP, Health services, Zimbabwe

## Abstract

**Background:**

In sub-Saharan Africa, adolescent girls and young women (AGYW) are at high risk of acquiring HIV. A growing number of sub-Saharan African countries are beginning to avail pre-exposure prophylaxis, or PrEP, but with limited success. Unpacking strategies to overcome barriers to the uptake of PrEP is critical to prevent HIV amongst AGYW. This article explores health professionals’ views and recommendations on what is required to increase uptake of PrEP.

**Methods:**

The study draws on interview data from 12 providers of HIV prevention services in eastern Zimbabwe. The healthcare providers were purposefully recruited from a mix of rural and urban health facilities offering PrEP. The interviews were transcribed and imported into NVivo 12 for thematic coding and network analysis.

**Results:**

Our analysis revealed six broad strategies and 15 concrete recommendations which detail the range of elements healthcare providers consider central for facilitating engagement with PrEP. The healthcare providers called for: (1) PrEP marketing campaigns; (2) youth-friendly services or corners; (3) improved PrEP delivery mechanisms; (4) improvements in PrEP treatment; (5) greater engagement with key stakeholders, including with young people themselves; and (6) elimination of costs associated with PrEP use. These recommendations exemplify an awareness amongst healthcare providers that PrEP access is contingent on a range of factors both inside and outside of the clinical setting.

**Conclusions:**

Healthcare providers are at the frontline of the HIV epidemic response. Their community-embeddedness, coupled with their interactions and encounters with AGYW, make them well positioned to articulate context-specific measures for improving access to PrEP. Importantly, the breadth of their recommendations suggests recognition of PrEP use as a complex social practice that requires integration of a combination of interventions, spanning biomedical, structural, and behavioural domains.

**Supplementary Information:**

The online version contains supplementary material available at 10.1186/s12879-022-07376-5.

## Introduction

Despite some HIV prevention successes, many countries and regions of the world are far off–track in reaching prevention targets set out by the United Nations General Assembly’s 2016 Political Declaration on Ending AIDS. In fact, more than 50 countries have experienced increases in HIV infection in recent years [1], and in 2020 an estimated 1.5 million people acquired HIV [2]. This is a long way short of the predicted target of fewer than 500,000 annual infections by 2020 [3]. The ambitious targets set by the United Nations reflect the hope and progress made to harness recent biomedical and health service successes in HIV treatment and the rapid scale-up of biomedical HIV prevention technologies, such as treatment as prevention (TasP), voluntary male medical circumcision (VMMC) and oral pre-exposure prophylaxis (PrEP). PrEP, which is the focus of this article, is a pill, which, when taken daily, significantly reduces the risk of HIV acquisition in the event of an exposure. PrEP is an efficacious HIV prevention method with trials showing that it can reduce risk of HIV acquisition by over 90% when taken consistently [4]. The World Health Organisation has expanded its recommendation on who should qualify for PrEP from key population groups to all people who are at substantial risk of HIV infection. As a consequence, PrEP now forms part of the repertoire of HIV prevention methods available to people in high HIV prevalence settings [5], expanding an already wide choice of prevention strategies that can be combined in different ways [6].

Adolescent girls and young women (AGYW) in sub-Saharan Africa (SSA) constitute a high-risk group for HIV infection. An estimated 25% of all new HIV infections in the region were amongst AGYW (aged 15–24), despite representing merely 10% of the population [2]. When looking at the 15–19 age group in SSA, a staggering six out of seven new HIV infections are acquired by adolescent girls [2]. Whilst these enormous inequalities require social protection responses across a range of HIV preventative domains, including access to education and socioeconomic safety nets and care and protection from the people around them [7], it also points to a need for HIV prevention technologies that appeal to AGYW. PrEP has been hailed for its promise to provide AGYW with user-control; yet uptake has been disappointing [8–10] and a growing body of literature has begun to unpack the broad spectrum of factors shaping AGYW’s uptake and continuous engagement. Much of this literature—quite rightly—draws on the perspectives and experiences of AGYW. This literature has identified a number of barriers to PrEP, including fears of how family, friends, partners and healthcare providers may react if they find out that an AGYW is using PrEP [11–14]. Studies have observed that these fears are rooted in a mix of sexuality and HIV-related stigmas [15, 16], and intersect with gender norms and ‘good girl’ notions, forcing AGYW to choose between the social risks of stigma or the risks of HIV acquisition [17]. Also practical challenges such as distances to health facilities [18] and finding the time and resources to attend routine screening and monitoring have been noted [11, 12]. More recently, a longitudinal study with AGYW in Cape Town, South Africa, has documented how intrinsic and extrinsic drivers of motivation to continue or discontinue with PrEP change over time, influenced by attitudes, practices and structures in the social fabric of AGYWs’ lives [19]. Although PrEP is a biomedical HIV prevention technology, requiring support and facilitation by healthcare providers [20], surprisingly little has been done to explore the experiences and perspectives of PrEP prescribers in sub-Saharan Africa. Against this background, and in our interest to inform efforts in the scale-up of PrEP in sub-Saharan Africa, we explore the perspectives, experiences, and recommendations of PrEP prescribers in low-resource and high HIV prevalence settings of eastern Zimbabwe.

## Methods

Data are drawn from a larger mixed-methods study exploring pathways to increase HIV risk perception and PrEP uptake amongst AGYW in east Zimbabwe [21]. Ethical approvals for the study were obtained from the Medical Research Council of Zimbabwe (REF: MRCZ/A/2243), the institutional review board of the Biomedical Research and Training Institute in Zimbabwe (REF: AP140/2017), and the Imperial College London Research Ethics Committee (REF: 17IC4160). Written informed consent was obtained from all participants with the agreement that their identities would be kept confidential. Pseudonyms have therefore been used throughout the study.

### Study location and participants

The qualitative arm of the study was conducted in two communities (Saksom and Watku) in Manicaland province in east Zimbabwe. Saksom is a high-density urban suburb while Watku is a rural village. The communities were selected for their urban and rural characteristics and for being low-resource high HIV prevalence settings. Average HIV prevalence in our broader study areas in Manicaland was 11% in 2015–16, down from over 25% at the end of the 1990s [22]. The qualitative explorations of the study included interviews and focus group discussions with a broad range of actors, including AGYW themselves, parents, community members and healthcare providers. In this article, we examine data from 12 in-depth and semi-structured interviews with healthcare providers recruited from two health facilities involved in the roll-out of PrEP—one from each setting. Six participants were recruited from the Saksom facility and six from the Watku facility. All of the healthcare providers were female trained nurses and they represent a broad range of work experiences including different primary care roles. A couple had been at the same facility for more than 20 years, whilst others had only worked at the current facilities for about a year after transferring from a different facility. A criterion for participant’s recruitment was that their role included providing HIV prevention services including PrEP to young people. None of the healthcare providers invited for an interview declined to participate. Upon recruitment, they were informed that there would be no financial rewards for their participation but that they would receive two large bars of soap as compensation for their time.

### Data collection and analysis

The study interviews were conducted in person, in a private room at the health facilities, between March and June 2019. Interviews lasted between 40 and 60 min with an average of 53 min. The interviews were conducted by three experienced and Shona-speaking qualitative researchers who were thoroughly trained in the study objectives and procedures. The researchers drew on a semi-structured topic guide covering themes such as healthcare providers’ perspectives on young people’s HIV risk awareness and HIV prevention behaviours—including uptake of HIV prevention methods—and their experiences of making HIV prevention services available to young people with specific questions focusing on PrEP. The topic guide also invited healthcare providers to share their top three recommendations for improving access to PrEP and other HIV prevention methods (See Additional file 1). All interviews were digitally recorded, transcribed, and translated into English. Protocols were in place to double-check the quality of transcription and translation. Interview transcripts were imported into NVivo 12, a software package for qualitative data management, coding, and analysis.

To provide an integrated view of cross-cutting themes in our data, we conducted a thematic analysis. Thematic analysis involves a subjective interpretation of data, searching for patterns of themes that emerge as important for the phenomenon under study which become categories for analysis [23]. To do this, we followed the thematic network analysis steps outlined by Attride-Stirling [24]. Similar to the technical set up of NVivo, her technique systematically organizes and indexes qualitative material into a web-like network of themes that unfold stories by moving from text to interpretation. In practice, this meant reading and re-reading the transcripts and inductively generating codes and what Attride-Stirling refers to as basic and organising themes (parent and grandparent nodes in NVivo). In this article, we focus on the 15 codes that contain text segments alluding to concrete recommendations made by the healthcare providers. We clustered the codes together into six basic themes, which we now proceed to present in turn, representing a mix of strategic areas of action that focus on either supply- or demand-side barriers.

## Results

PrEP was made available to AGYW from the participating health facilities in February 2019, with the first users being reported at the beginning of March 2019. As the interviews were conducted between the end of March and June, 2019, PrEP delivery was still new to our participants, and they were still forming their *modus operandi*. Nurses, pharmacy and laboratory department representatives had undergone a two-day training course on PrEP delivery in January 2019. The training focused on equipping the healthcare providers with the knowledge and skills to initiate and manage clients on PrEP. Specifically, they were trained on how to identify eligible candidates for PrEP which included both testing HIV-negative and HIV risk assessments. They were also trained on how to counsel and monitor PrEP users; both to sustain adherence and to ensure clients returned for follow-up visits. We analyse their early experiences and recommendations for making PrEP accessible, commencing first with their recommendations to improve the supply of PrEP services, followed by their recommendations for PrEP demand creation.

### Need to strengthen the human resource capacity of health services to deliver PrEP

There was a general perception amongst the healthcare providers that their human resource capacity to make PrEP services available in a timely manner was inadequate, which may impact on AGYW’s continued engagement with this prevention technology. This includes both how quickly AGYW are served and the amount of time they spend with the healthcare provider. Tendai highlights two pressing issues. One is that no healthcare provider at their facility works solely on PrEP delivery; the other is that AGYW need services right away.

People who offer PrEP have other commitments in the health facility. There is need to have someone dedicated to PrEP and nothing else. For example, someone like me may be busy helping someone deliver their baby, delaying someone seeking PrEP. When someone gets up and decides to look for PrEP, it would be helpful for them to get the services right away. Tendai

Tendai argues that the only way to avail timely services for AGYW is to have someone readily available. Tendai also recognises the difficult choice of AGYW to seek PrEP, and underlines the importance of honouring this decision by being readily available. Chipo, in her account of how AGYWs have to wait for a suitably qualified healthcare provider to counsel them about PrEP, expressed a similar sentiment by saying “You will take the client’s time. This is bad.”

Their limited human resource capacity restricts the number of qualified people available to administer PrEP and therefore affects the amount of time they can spend with each client. Blessing explains how she, in addition to offering PrEP, is tasked with other nursing duties and struggles to make available the time required to counsel and support AGYW seeking PrEP.

This one is a big challenge because counselling for PrEP doesn’t take only 5 minutes, it needs 1 hour and so on and you have other clients you would like to serve. This actually acts as a drawback because some people would come wholeheartedly wanting to discuss a certain issue and wanting to know more about other issues. You don’t have enough time to talk to that person so the issue of human resources is the main reason why a person might return without being satisfied with the services and getting assisted when she comes. Blessing

Tendai ends her account of this challenge by highlighting the need for nurse training programmes to integrate PrEP delivery into their curricula so that more nurses can step in and support PrEP delivery to a generation of inquisitive youth.

There is need to have this in the curriculum so that lecturers go and address this. Kids nowadays want to know so much and they have many questions to ask. Tendai

Tendai, Chipo and Blessing’s accounts all point to a need to treat AGYW as customers whose uptake and continued engagement with PrEP requires them to make HIV prevention services available in a timely fashion; something that requires investments in human resource capacity for PrEP services to be delivered effectively.

### Need for improvements in PrEP treatment and delivery

The nurses also expressed concerns about PrEP treatment and delivery, linking the size of the pill, (anticipated) side-effects, and delivery of PrEP through HIV treatment clinics/wards to possible disengagement with PrEP.

Nurses, often based on their experiences of distributing and taking post-exposure prophylaxis (PEP) pills, were sceptical about AGYW actually taking the PrEP pills on a daily basis—scepticism that was rooted in the pill size:

I don’t know if researchers are able to find something that is better because this tablet, even holding it, is hectic. We don’t even know if the AGYW we gave PrEP went away and took their medicines because that pill is big. Even when you hold it in your hands. It’s too big. I doubt they finish the course because those taking PEP, even nurses who know the importance of taking drugs, don’t finish PEP. Now imagine an ordinary person being expected to be consistent in taking PrEP. Tendai

Tendai makes a direct appeal to researchers; calling for smaller pill sizes. A number of healthcare providers articulated the need to reduce or address side-effects associated with PrEP. This was often followed by detailed descriptions of their experiences of how AGYW may physically react to PrEP treatment.

Some will feel dizziness, some will vomit, and some will struggle to sleep. These are some of the problems which people encounter. Some would have rashes. So this will become a problem when she starts using PrEP. Blessing

One nurse, in a conversation about AGYWs PrEP use, alluded to how anticipation of side-effects may constitute a significant worry and deterrent to engaging with PrEP: “They are worried about the side effects of PrEP. That is their biggest worry.” The conversation about side-effects was often accompanied with reflections on how AGYW may think about PrEP and it was not uncommon for a healthcare provider to speculate that anticipation of side-effects heightened AGYWs pre-existing scepticism about enrolling onto a treatment programme when healthy. For many healthcare providers, addressing possible side-effects, as well as associated rumours, are key to AGYW’s engagement with PrEP.

How and where PrEP pills were administered also emerged as problematic. In this context, PrEP is accessed through sexual health clinics or wards, just like antiretroviral drugs for HIV treatment. The healthcare providers talked about how some AGYW, because of their fear of being associated with an HIV-positive status, would avoid visiting the sexual health clinic. This was said to be heightened by the time and human resource constraints discussed above, as AGYW would likely find themselves waiting on benches at the sexual health clinic, on public display, and next to people waiting to pick up their HIV treatment. Continued pervasive stigma against people living with HIV and youth sexuality made this an undesirable scenario, affecting their disengagement with PrEP, something we have discussed in detail elsewhere [author ref.]. Because many healthcare providers perceived PrEP-related stigma as being a problem, they recommended that PrEP pills should be administered in containers that disassociate them from HIV treatment, as exemplified by Alice:

PrEP should be dispensed in another container. They don’t want it to be dispensed in its original containers which are easily identified and associated with HIV treatment. Alice

The healthcare providers participating in this study see many challenges in the delivery of PrEP—ranging from the pill itself and its possible side-effects to how it is administered at health facility level—but also present a number of recommendations to overcome these challenges. Related to the stigma associated with PrEP, we will now elaborate on their call for more youth-friendly services.

### Need for youth-friendly PrEP services

Possibly due to the training they had received, our participants were fairly self-critical of how they, as a group of healthcare providers, interacted with AGYW. Healthcare providers from the rural community in particular were aware of how their community-embeddedness affected their perceptions of AGYW’s sexuality. Some spoke openly about their difficulties in advising young girls to use PrEP when they themselves think they are too young to engage in sex. Anika has developed a recommendation for herself; namely “give them the appropriate information that they have come to the health facility for without looking at their age”. Tendai articulates a similar recommendation, calling for non-judgmental PrEP services:

The other thing that demotivates the children to access PrEP services is that, when they get to the health facility gate and ask for PrEP, they will be met by someone older, like me, who will judge them for being sexually active at that age, when in fact they should be getting assisted. So yes, health staff can be demotivating. When children come looking for PrEP there is no need to start asking why they are sexually active, just focus on talking them through PrEP. Tendai

It is noteworthy that the participants began referring to AGYW as children the moment they began to critically reflect and talk about how they pass judgement on AGYW’s sexuality. Healthcare providers are not alone in passing judgement; so do members of the broader community who may stumble across AGYW at or near the sexual health clinic. One nurse said: “once you are seen going there you will be judged.” As discussed earlier, this is likely to deter a number of AGYW from accessing PrEP. A number of participants therefore recommended that PrEP be provided through more neutral and anonymous channels such as outpatient clinics.

Those who need PrEP should be separated from those accessing antiretroviral drugs for treatment. PrEP could be distributed at the outpatient clinic instead of the opportunistic infections department, simply because some AGYW will be afraid of stigmatisation. Blessing

A number of other suggestions pertained to developing more youth-friendly services. Suggestions ranged from having clinics or corners within the health facility, designated for young people, or employing a cadre of young healthcare providers who can relate to young people in a different way. Our study participants recognised a mismatch between how PrEP services are delivered and the lived realities of AGYW. One nurse, Louise, call for a rescheduling of the PrEP service to cater for the availability of adolescents who spend most of their daytime in school.

The challenges present may include the fact that most adolescents will be school-going age so there should be time set aside for them so that services are scheduled to be offered at a certain time catering for those adolescents who won’t make time because they are going to school at all times. Louise

While our participants were not short of recommendations to overcome barriers related to how they and the way PrEP services were organised and delivered at their health facilities, they also recognised that many social and community-level barriers persist that affect AGYW’s demand for PrEP. We will now outline their recommendations for accelerating AGYW’s demand for PrEP.

### Need for PrEP awareness campaigns

Our participants spoke unanimously about the need for PrEP awareness campaigns. They called for campaigns, like those targeting young men with VMMC, to make young women aware of PrEP. Chipo talked extensively about the role and need for schools to educate AGYW and young men about PrEP:

Campaigns about PrEP should be done in the community and in schools. I believe nothing is said about PrEP in schools, yet that’s where most young people who do not come for PrEP are found. We know that they will be engaging in sexual activities but they don’t come for PrEP. Why? They don’t have information about PrEP. If we have campaigns in the community and children are taught about PrEP in schools, this will help us in HIV prevention. Chipo

Chipo concludes by noting that school-based campaigns about PrEP should not stand alone; rather they should be accompanied with campaigns in the community, reaching parents, neighbours and other key stakeholders in order to reduce some of the PrEP-related stigma outlined earlier. There was also a general agreement that the campaigns should include information about PrEP that debunks myths about PrEP. Clear communication about possible side-effects was stressed as being particularly important, as they perceived young people to have misconstrued understandings of the risks of using PrEP, amplified by ‘fake news’ on the internet.

So now when it comes to youths, they now go on the Internet a lot and they can go and Google the side-effects that these tablets have, and hear about worst case scenarios. Tendai

### Need to proactively engage with AGYW

While all the healthcare providers talked about the need for greater awareness-raising about PrEP, a number of them also highlighted that it was important that this health information did not just come from them or other public health authorities. They actively encouraged PrEP users to come forward, by coming together, to show their communities who they are and help educate people in their communities:

Alright, I think we should form support groups with the people taking PrEP so that people can see what kind of people are taking PrEP. I believe that someone who is actively involved with PrEP must educate people in their community; only then will people understand what it means to be on PrEP. Melinda

Melinda believes that much of the stigma and misconceptions surrounding PrEP can be challenged if more people come forward; establishing support groups for PrEP users is one way to start mobilising such a change and encouraging demand for PrEP.

Another suggestion that emerged from the interviews reflects an entirely different form of engagement; one that circumvents demand creation and involves directly seeking out AGYW at risk and bringing them to the health facility. Rather than encouraging them to make use of PrEP services, a small group of healthcare providers reflected on some of the more aggressive initiatives to increase uptake of VMMC, and suggested the same should apply to PrEP:

When they go out and try to identify the young women at risk I think it’s better to like when they have identified they shouldn’t just be given a referral letter, I think they should try to… like what VMMC does, they go and collect the clients and bring them to the site. I think they should just bring the clients and we give PrEP. I think that would do. Alice

This however may be seen as too coercive and possibly counter-productive for AGYW, highlighting the need for further consultation and community engagement on possibly ways to reach AGYW. One of the arguments put forward for collecting and bringing AGYW to the health facilities relates to the costs of seeking PrEP. This brings us to the final recommendation.

### Need to eliminate costs associated with PrEP uptake

In many of our interviews, healthcare providers discussed how the different costs associated with PrEP use served as a barrier to uptake. Resonating with the aforementioned comment about the need to collect AGYW, was a recommendation to reimburse bus fares. Such suggestions emerged from discussions about how adolescent girls who live in poverty, and who may not even go to school, will not be in a position to ask their caregivers about money for bus fares.

… some are even failing to go to school; others even being given bus fare to come and collect their medication it’s a problem because their caregiver will tell them that they don’t have the cash to give them as bus fare. Melinda

The recommendation to reimburse bus fares came predominantly from healthcare providersprovider in the rural setting where distances are greater and public transport may be required. In addition to potential transport costs, the healthcare providers outlined health facility costs associated with PrEP uptake which they said ought to be eliminated. Health facilities in the study districts require a minimum charge of USD 1 or the equivalent Zimbabwe dollars for patients aged 5–64 years for consultation before being referred to the relevant department to get the service required. For AGYW, there were—in principal—no further costs associated with PrEP. Yet, this small fee was noted as a barrier preventing some AGYW from accessing PrEP services:

I think that is the biggest challenge and also someone may not have the small service fee for consultation let’s say they wanted to be seen and talk about and someone may not have the consultation fee. Alice

**Fig. 1:**
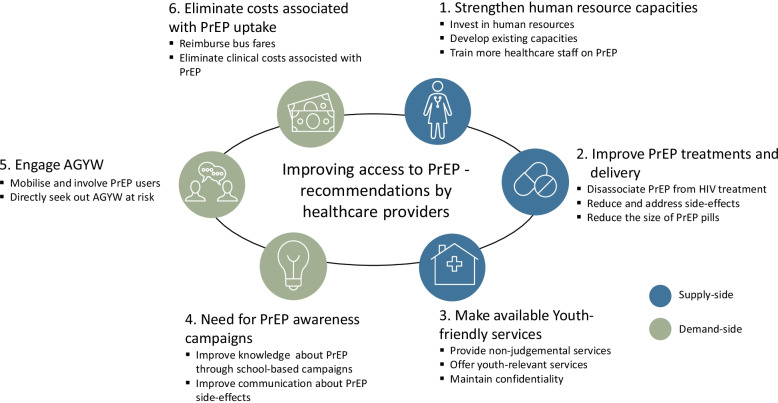
Healthcare provider recommendations for improving access to PrEP

Our analysis revealed 15 concrete recommendations to improve access to, and the quality of, PrEP delivery. Figure 1 visually illustrates these recommendations, which span six strategic focus areas (the headings of our result section), covering both supply- and demand-side interventions.

## Discussion

There is little published material reporting on the experiences and perspectives of healthcare providers involved with implementation of PrEP delivery in SSA. In an attempt to begin to address this gap, and to inform policy and practice in the scale-up of PrEP, we invited healthcare providers in eastern Zimbabwe to share their experiences of prescribing PrEP and recommendations for overcoming barriers. Many of the recommendations corroborate and further detail early evidence from PrEP implementation projects in SSA.

From the supply-side perspective, the healthcare providers in our study fully recognised their own constraints and cultural biases in prescribing PrEP to AGYW. This is not only in agreement with our own experiences and observations in the trial, with for instance nurses talking AGYW out of taking up PrEP, but also resonates with findings from a study in Tanzania, where healthcare providers, because of their attitudes towards AGYW sexuality, caught themselves providing stigmatizing and discriminatory care [25]. As a result, our participants advocated strongly for both youth-friendly services and a strengthening of human resource capacities to deliver PrEP in non-judgemental and safe ways. They also called for a disassociation of PrEP from HIV treatment. These recommendations resonate with findings from a recent qualitative study with AGYW in South Africa and Kenya, which found adolescent-friendly and integrated sexual and reproductive health services to facilitate PrEP uptake [26]. In fact, a review of early lessons from PrEP demonstration projects in SSA found PrEP initiation and impact amongst AGYW to be greater in projects that integrated PrEP into youth-friendly clinics that combined a range of different sexual and reproductive health services [10]. Recognising their lack of financial resources and trained staff to provide timely and responsive care to AGYW, challenges also identified by healthcare providers in Kenya [11], all the healthcare providers in our study recommended further investments in the healthcare cadre involved in PrEP provision.

Unlike in any previous work with PrEP prescribers in SSA, the healthcare providers in our study also appealed to biomedical innovators of PrEP. They raised concerns about the size of the pill, potential side-effects, and the packaging of PrEP, factors they expressed as barriers for AGYW’s uptake and continuous engagement with PrEP. Similar concerns have been raised by AGYW themselves elsewhere in Zimbabwe, where Gombe and associates [12] found some AGYW to indicate that the size of PrEP pills might challenge adherence. They also found side-effects to be an issue; albeit an issue AGYW learnt to overcome. A focus group study with AGYW in Kenya, however, found side-effects to constitute a barrier to PrEP uptake and engagement [11]. The study also noted issues related to the packaging of PrEP. Most participants reported a transference of stigma from anti-retroviral therapy to the PrEP pill because of their similar appearance and packaging. As observed amongst AGYW in both South Africa [14] and Zimbabwe [15], difficulties in concealing the pills challenge non-disclosure. Some providers are beginning to respond to this concern, such as the ‘V’ initiative in southern Africa, where PrEP tablets are being repackaged in containers that look like lip-gloss (www.conrad.org/launchingv).

From a demand-side perspective, the healthcare providers in our study fully recognised that the costs associated with PrEP use may be prohibitive for some of the most vulnerable AGYW. They recommended elimination of costs associated with accessing PrEP, such as any expenses incurred by the health facility, and reimbursement of transport costs. A number of studies with PrEP users in sub-Saharan African contexts note lack of funds for transport and poverty as barriers to PrEP [12, 27]—supporting this recommendation. Whilst costs may be a barrier to existing demand, the healthcare providers in our study also alluded to a lack of PrEP demand and unmet needs. They proposed four concrete recommendations for demand-creation, which warrant engagement with communication strategies such as the PrEP Communications Accelerator proposed by Schwartz et al. [28]. First of all, they call for PrEP awareness campaigns that both make AGYW aware of PrEP and challenge prevailing ‘fake news’ and misconceptions about PrEP and its side-effects. Importantly, they note the need to reach and educate everyone at community-level, and not just AGYW. Second, they call for greater engagement with AGYW in demand-creation. In agreement with observations amongst AGYW in Kenya and South Africa [26], they point towards the need to identify peers who can come forward and share their experiences of being on PrEP, and serve as role models or PrEP ambassadors. The healthcare providers also noted that they could play a more active role in identifying and recruiting AGYW to PrEP. They talked about physically fetching AGYW from the communities. Rather than fetching AGYW and bringing them to the health facility, healthcare providers can perhaps do what has been observed elsewhere in Zimbabwe, namely take an active role in reaching out to, and making local AGYW aware of PrEP [12].

There are constraints to interpretation of our findings. One, our data were produced in the early days of PrEP roll-out in Manicaland, affecting the comparability and transferability of our findings to settings where PrEP roll-out is more mature. The dynamic implementation of initiatives to improve PrEP service provisions are likely to continually shape the experiences and types of recommendations coming from healthcare providers. Similarly, whilst our rural and urban findings were broadly similar, our findings are highly dependent on our country context, which is characterised by particular socio-cultural norms and PrEP service delivery structures. Our study also only focuses on the perspectives of healthcare providers and does not include the voices of the key population: adolescent girls and young women. Whilst we report on their perspectives in other articles [17, 29], future research must consider the voices of AGYW when devising recommendations that focus on improving their engagement with PrEP. How data was produced and interpreted for this article reflects the different positions of the researchers involved. For instance, the interviews constitute interactionist encounters between the local researchers and the healthcare providers. The healthcare providers may have been subjected to what Scott and Lyman [30] refer to as a ‘valuative inquiry’, where they felt compelled to explain and justify the unanticipated or untoward behaviours of AGYW. This interactionist context may have contributed to specific types of accounts and explanations for poor PrEP uptake, including the emerging recommendations. Furthermore, our interpretations reflect our interdisciplinary backgrounds, collaborative approach to analysis, as well as our pragmatic interest to produce knowledge that is actionable.

## Conclusions

Healthcare providers are at the frontline of the HIV epidemic and the present study is one of the first to qualitatively explore and analyse their particular perspectives and recommendations to improve PrEP uptake and quality of care in a sub-Saharan African setting. The study findings highlight that healthcare providers are more than prepared to play a role in promoting PrEP use, but also emphasize that key strategies to improve PrEP uptake and quality of care are to invest in the training and competency-building of healthcare providers, and to develop innovative and youth-friendly approaches in the demand-creation and delivery of PrEP. The study exemplifies an awareness amongst healthcare providers that PrEP access is contingent on a range of factors both inside and outside of the clinical setting. The breadth of their recommendations suggests recognition of PrEP use as a complex social practice that requires the integration of a combination of interventions, spanning biomedical, structural, and behavioural domains [31].

It is critical for policy makers and health managers to consider the perspectives of healthcare providers. Their community-embeddedness, coupled with their interactions and encounters with AGYW, make them well positioned to articulate context-specific measures for improving access to PrEP. Based on real-time learning from this study, co-author, PMM, together with colleagues from the Zimbabwe Ministry of Health and Child Care implemented PrEP service awareness and promotion activities in Mutasa and Nyanga districts. Focusing on recommendations 4 and 5 (Fig. 1), they recruited and trained PrEP mobilisers, who adopted different strategies to create awareness of local PrEP services. The strategies included gatherings for AGYW, Q&A WhatsApp groups for AGYW, Walk-in sessions, and door-to-door outreach. Through our collaboration with Zimbabwe Ministry of Health and Child Care and Zimbabwe National AIDS Council at national, provincial and district levels, findings from this study, and our other surveillance activities, are fed back to national and local policy-makers.

## Supplementary Information

**Additional file 1.** Topic guide for in-depth interviews with health service providers.

## Data Availability

The interview guide is included here as a Supplementary file. The data generated and analysed for the current study are not publicly available, as participants only consented to make their data available for research and education purposes. Data are available from the corresponding author on reasonable request.

## Abbreviations

AGYW: Adolescent girls and young women
HIV: Human immunodeficiency virus
IDI: In-depth individual interview
PEP: Post-exposure prophylaxis
PrEP: Pre-exposure prophylaxis
SSA: Sub-Saharan Africa
