# Supplementary material for: Improving access to pre-exposure prophylaxis for adolescent girls and young women: recommendations from healthcare providers in eastern Zimbabwe
Source: BMC Infect Dis. 2022 Apr 23;22:399. doi: 10.1186/s12879-022-07376-5 (PMC9035262; doi:10.1186/s12879-022-07376-5)
Supplement: Supplementary file 1 — Additional file 1. Topic guide for in-depth interviews with health service providers. [file 12879_2022_7376_MOESM1_ESM.docx]

**Topic guide for in-depth interviews with health service providers**

| **QUESTION AND PROBES** | **NOTES/RATIONALE** |
| --- | --- |
| **Background/icebreakers** |  |
| I’d like to begin by asking some questions about you, your job, and what you do from day to day:   - Please tell me a bit about yourself: where do you come from? Do you live in this community? - How long have you been working at this clinic? What were you doing before working here? - What specific type of healthcare do you provide? Have you always had this role? | To get to know the participant a little better. You should bear these responses in mind during the rest of the interview. This section is also designed to help the respondent feel at ease. |
| **HIV risk awareness** |  |
| We would like to hear about how HIV has affected this community, and what this means for young people and their relationships:   - Are young people concerned about HIV? Are there differences between young women and men? - When young people have sex or start a new relationship, do you think they consider the risk of HIV? Please explain. - What should be done to improve young people’s awareness of HIV risk? - What is the best way to disseminate information about HIV risk? | To understand service provider’s perceptions of young people’s awareness of HIV, and the implications of HIV on their relationships and sexuality |
| **HIV prevention behaviours in your community** |  |
| We’re interested in hearing about HIV prevention behaviours in your community:   - What should young people do to avoid HIV? - What are some of the challenges for young people to avoid HIV? - Are these challenges the same for young women and men? If not, please explain. | To understand what young people in the community do to avoid HIV |
| **HIV prevention methods** |  |
| We’d like to ask some questions about why young people do or don’t take advantage of HIV prevention methods available from your clinic**.**   - What HIV prevention services do you offer in your clinic? - What kind of HIV services do young people ask for in your clinic? - What kinds of young people come for HIV prevention services?  Probe: young/old, single/married, rich/poor, etc.? - What motivates young people to make use of HIV prevention service?   Probe: Their decision? Pressure by health workers? Pressure by others? Partners? parents?   - What kind of challenges do young people face in accessing HIV prevention services? Probe: Do you think this has been changing over time? - What do you think is the best way to support young people access HIV prevention services?  Probe: school-based counselling, provider-initiated, advice from parents, partner testing, HIV testing etc? - How do young people feel when they come to the clinic and seek out HIV prevention services? Probe: can you give me some examples? - Do young people feel free talk about HIV and sex?   Probe: With who? Partner? Friends and family? How did they react?   - How do you make sure that young people continue to make use of HIV prevention services? - What challenges do you face in providing high quality HIV prevention services? What can you do better? | To understand what prevention methods are available in the community and challenges in accessing and using these methods. |
| **Pre-exposure prophylaxis** |  |
| Let’s talk about PrEP:   - How might PrEP be viewed in your community? And amongst young men and young women? - Can you describe what kind of young people come for PrEP? What kind of young people do not come for PrEP? What motivates or demotivates young people from accessing PrEP? - What do these young people generally know and feel about PrEP?   Probe: Do they understand how it works? The benefits? Are the men scared?   - What kinds of challenges do young people face in coming for PrEP in your clinic? - What worries or concerns do they have about taking PrEP?   Probe: secrecy, gossip, food, partners   - How do you feel about PrEP services on offer here? Any recommendations for what could be changed to motivate more young people to make use of PrEP? - What kinds of other health problems do patients suffer from when they start PrEP? - What do you do when you see signs of poor adherence? - What do you or others at the clinic do to encourage young people to keep taking PrEP? Does this help? - What kind of young people stop taking their PrEP? - What makes a ‘good’ HIV prevention service user?   Probe: Why do you say this? Can you give me an example?   - What makes a ‘challenging’ HIV prevention service user?   Probe: Why do you say this? Can you give me an example?   - Are there special services for different groups of people? MSM? Adolescents? Sex workers? Couples? Women? Can you tell me about them? What are the challenges? What works well? - Do you feel equipped to offer PrEP services? - What does this clinic do well in providing PrEP services? - What does this clinic do poorly? - What challenges do you face in providing PrEP? | To understand what people think about about PrEP in the community and challenges to service delivery |
| **Recommendations** |  |
| Finally, I’d like to ask for your recommendations to help improve the uptake of HIV prevention services in this community.   - What three things do you think should happen in this community to improve the uptake of HIV prevention methods amongst young people in this community? | To understand what they feel are priorities for change in the community and in health services. |
| **Wrap up** |  |
| Finally:   - Is there anything else that you’d like to discuss today? - Are there any questions that you’d like to ask me about anything that we have discussed today? |  |
